# Acknowledgment to the Reviewers of *Non-Coding RNA* in 2022

**DOI:** 10.3390/ncrna9010008

**Published:** 2023-01-13

**Authors:** Non-Coding RNA Editorial Office

**Affiliations:** MDPI AG, St. Alban-Anlage 66, 4052 Basel, Switzerland

High-quality academic publishing is built on rigorous peer review. *Non-Coding RNA* was able to uphold its high standards for published papers due to the outstanding efforts of our reviewers. Thanks to the efforts of our reviewers in 2022, the median time to first decision was 15.5 days and the median time to publication was 43 days. Regardless of whether the articles they examined were ultimately published, the editors would like to express their appreciation and thank the following reviewers for the time and dedication that they have shown *Non-Coding RNA*:
Abhishek DeyKarina VargovaAgnieszka BelterKarla RubioAgnieszka Dzikiewicz-KrawczykKi Hyun NamAgnieszka Jagiello-GruszfeldKongpan LiAlan DombkowskiKrishna Chaitanya SuddalaAleksandra WardzyńskaKrystyna Mazan-MamczarzAlexander PasternakKrzysztof FlisikowskiAlexandra PeregrinaKunhua SongAlexandra Peregrina LavínKunzhe DongAmit SinghLeon N. SchulteAnanthanarayanan KumarLiming ChenAndreas WernerLing YangAndrzej KoniecznyLuke SelthAnne BickerMagdalena BogutaAnnunziata MauroMahsa M. AmoliAntonino FiannacaMaria Alexandra PapadimitriouAnurag SharmaMaria Eugenia ArizaAria BaniahmadMaria Isabel Nogueira CanoArturo BonomettiMaria PoptsovaArun Kumar Hanumana Gouda PatilMarina CristoderoAssam El-OstaMasayuki NashimotoBård KarlsenMaxime WeryBartlomiej KrazinskiMd Noushad JavedBenjamin RocheMichael JacksonBing YangMichael R. LadomeryBodhisattwa BanerjeeMichelle WattsByron BaronMinakshi GandhiCarlos Alberto Moreira-FilhoMinjie ZhangCarolina Susana CerrudoMirolyuba IlievaCatherine Vénien-BryanMisbah KhanChang LiMohammed GrawishChristian LaboisseMohan BabuChristine HappelMuller FabbriCindy KörnerMuthukumar RamanathanConstain H. SalamancaNik TsotakosDaniela TavernaNorman H. L. ChiuDavide MarangonNuo-Min LiEduardo Andrés-LeónOrazio FortunatoEduardo LarribaPablo SmircichEduardo López-UrrutiaPadet SiriyasatienElena S. Martens-UzunovaPanagiotis PapoutsoglouEmiliano FratiniPeng ShaoEstanis NavarroPeter UnrauEugenio MorelliPrajish IyerEun-Ok LeeQamar U. ZamanFelipe Cesar Ferrarezi BeckedorffQuoc Thang PhamFlora BrozziRahaba MarimaFolkert J. Van WervenRahul Shubhra MandalFrancisco EnguitaRamin FarahaniFu RuiRaquel EchavarriaGayatri ArunRussell HamiltonGracjan MichlewskiSabina SevcikovaGrasiele SausenSaeid GhorbianHe ZhangSaikat BoliarHisashi MeraSantosh UpadhyayHua-Sheng ChiuSebastián Alexis VishnopolskaHung-Ming LamShamseddin AhmadiIlaria GuerrieroShizuka UchidaImadeldin ElfakiShowkat DarIsabel S. Naarmann-de VriesSohini ChakrabortyIvan B. FilippenkovSouvick RoyJan NovákStephan BernhartJens Claus HahneSushil Kumar ShakyawarJesús Adrián LópezSusmitnarayan ChaudhuryJiali YuTallulah AndrewsJoanna SolichUfuk DegirmenciJohn MattickVishal ChavdaKaifu ChenXufeng HuangKamariah IbrahimYves HenryKankadeb MishraZeng-Hong Wu


